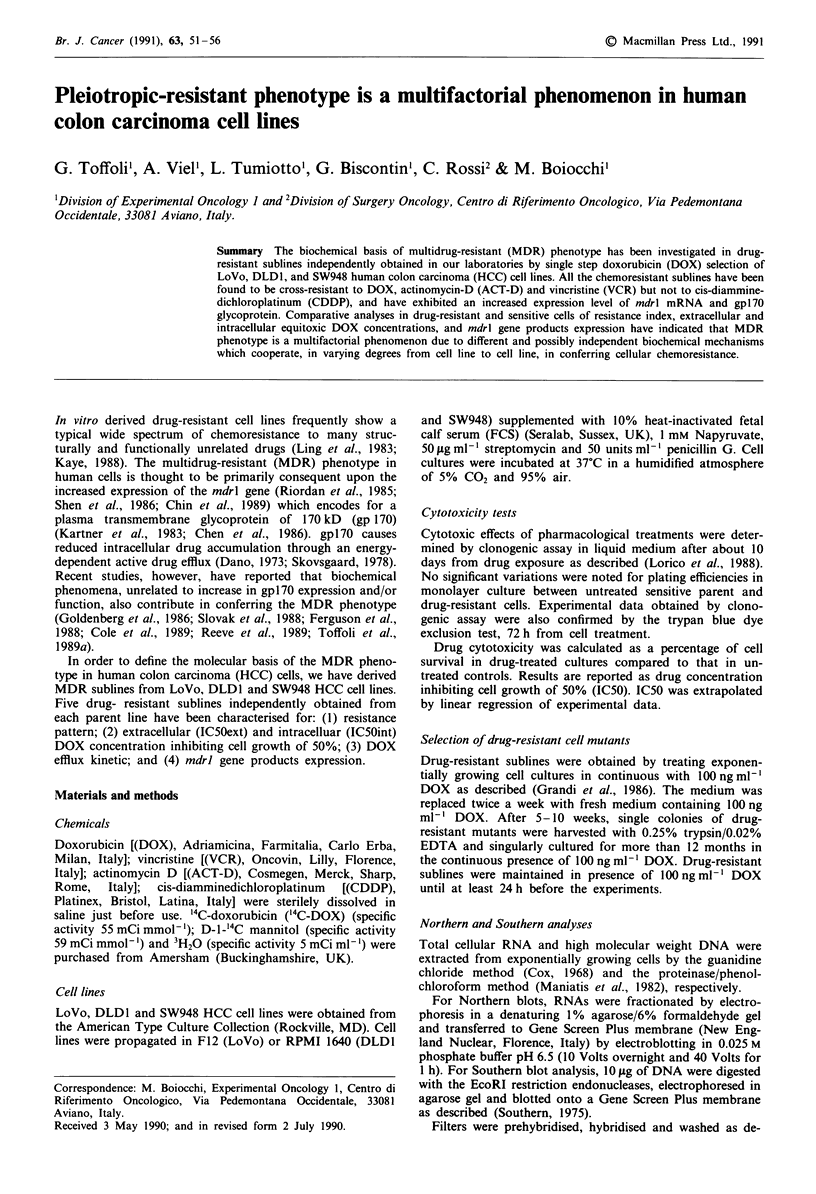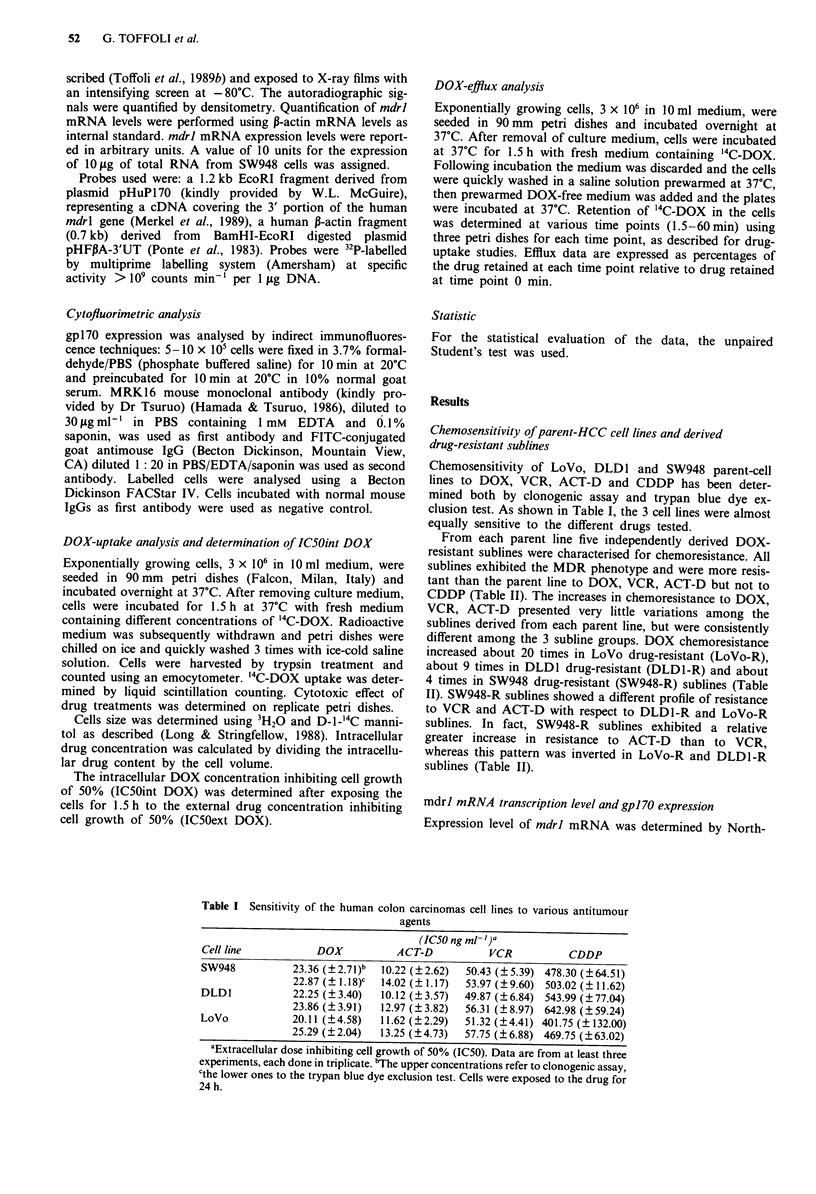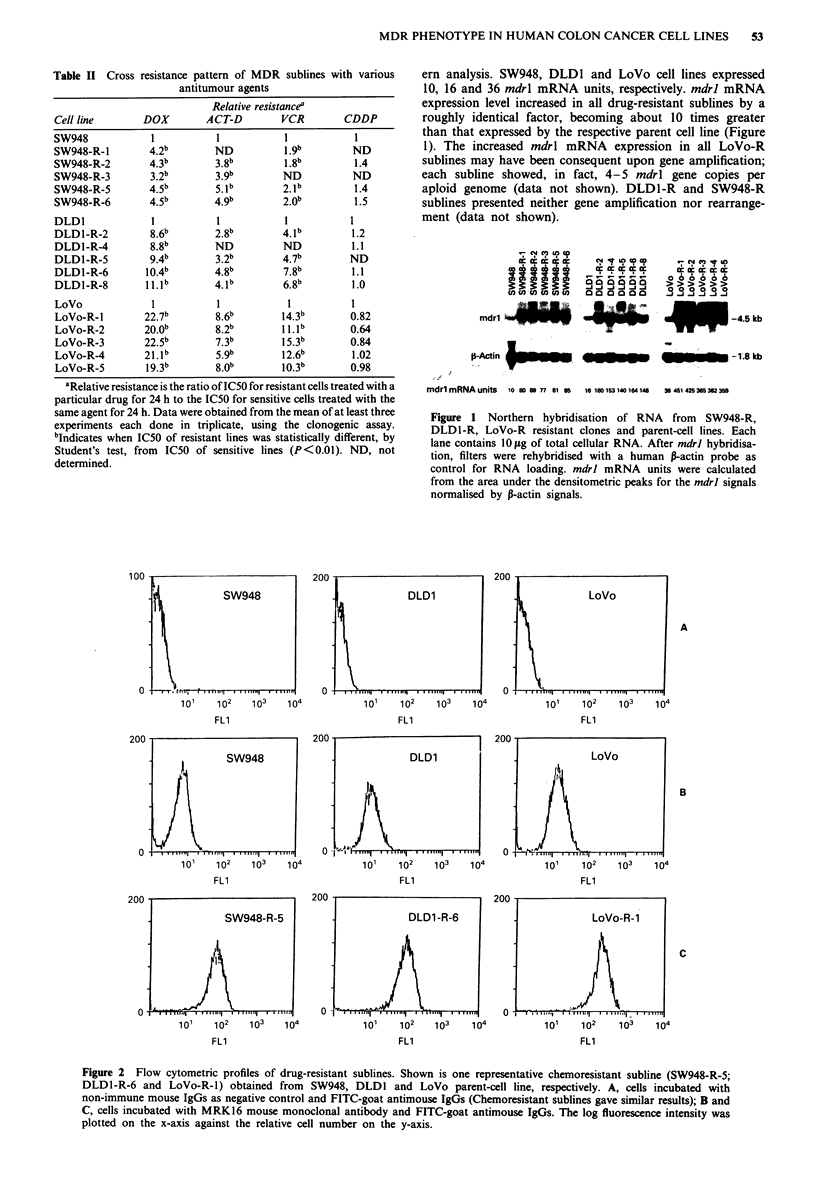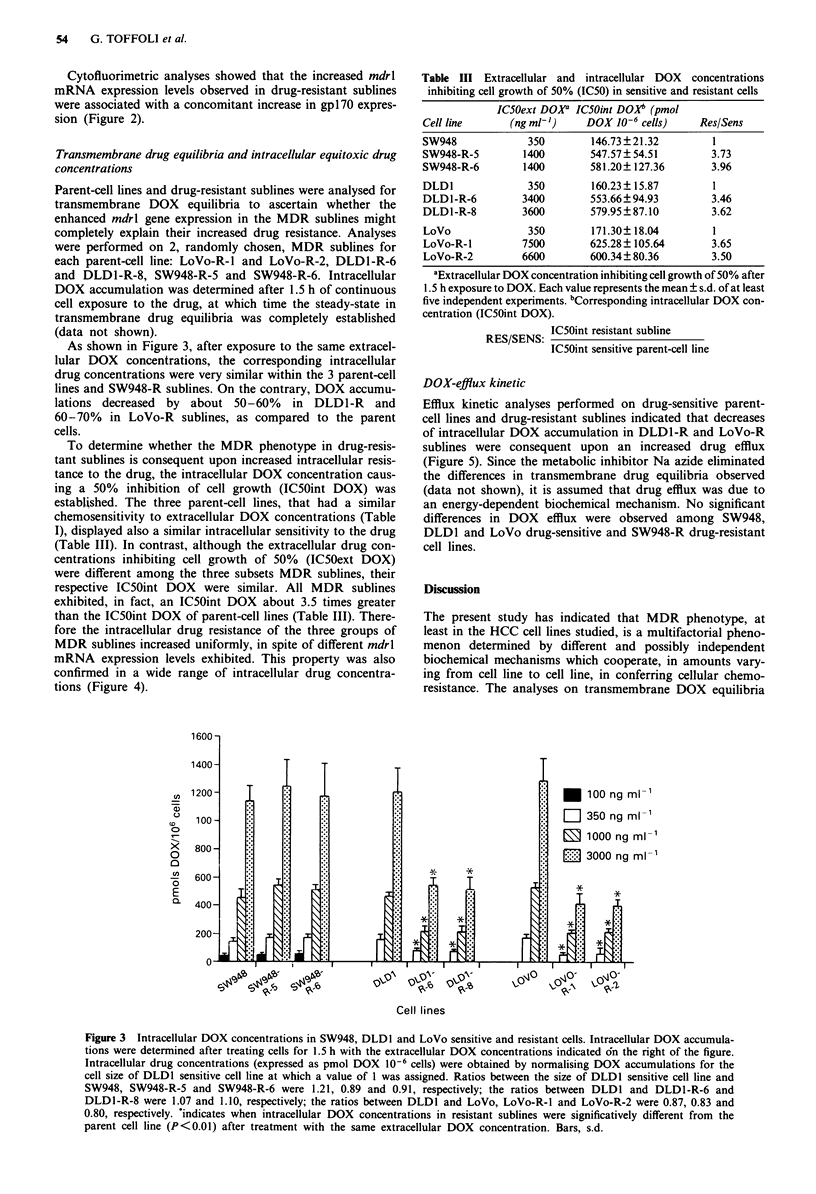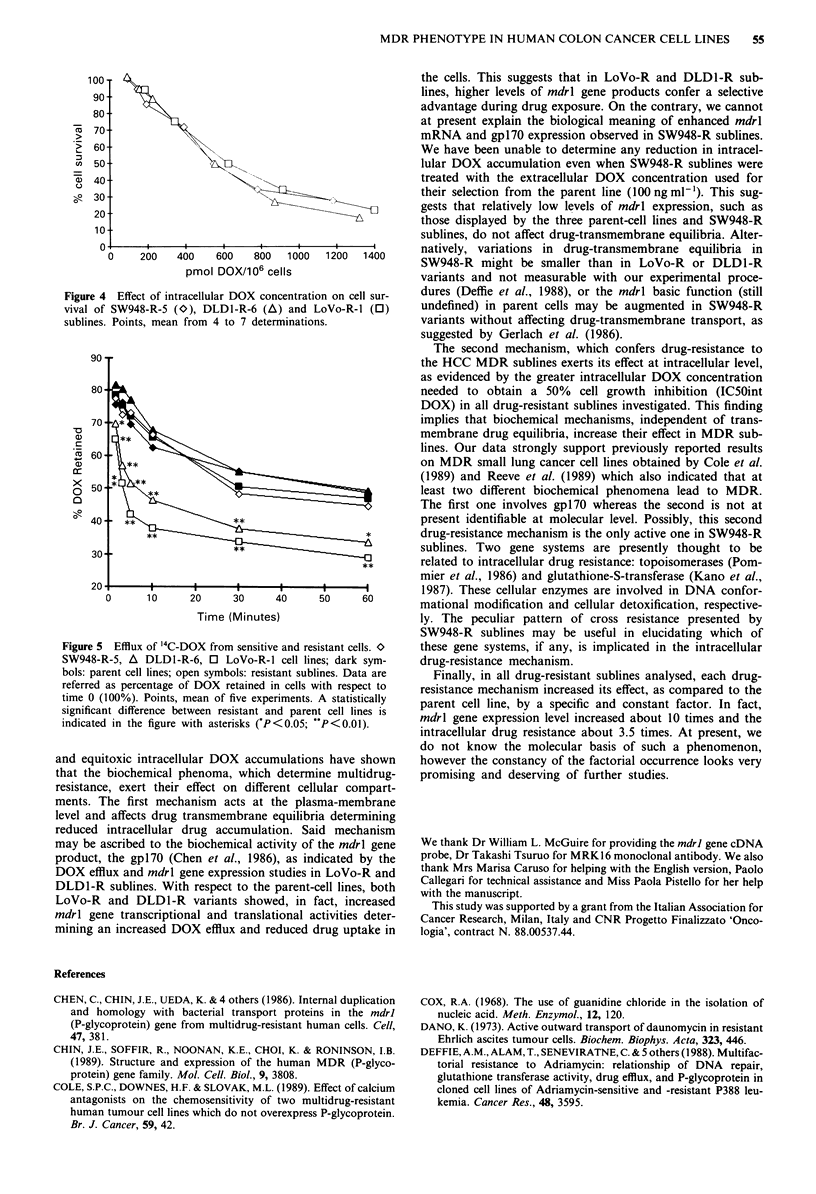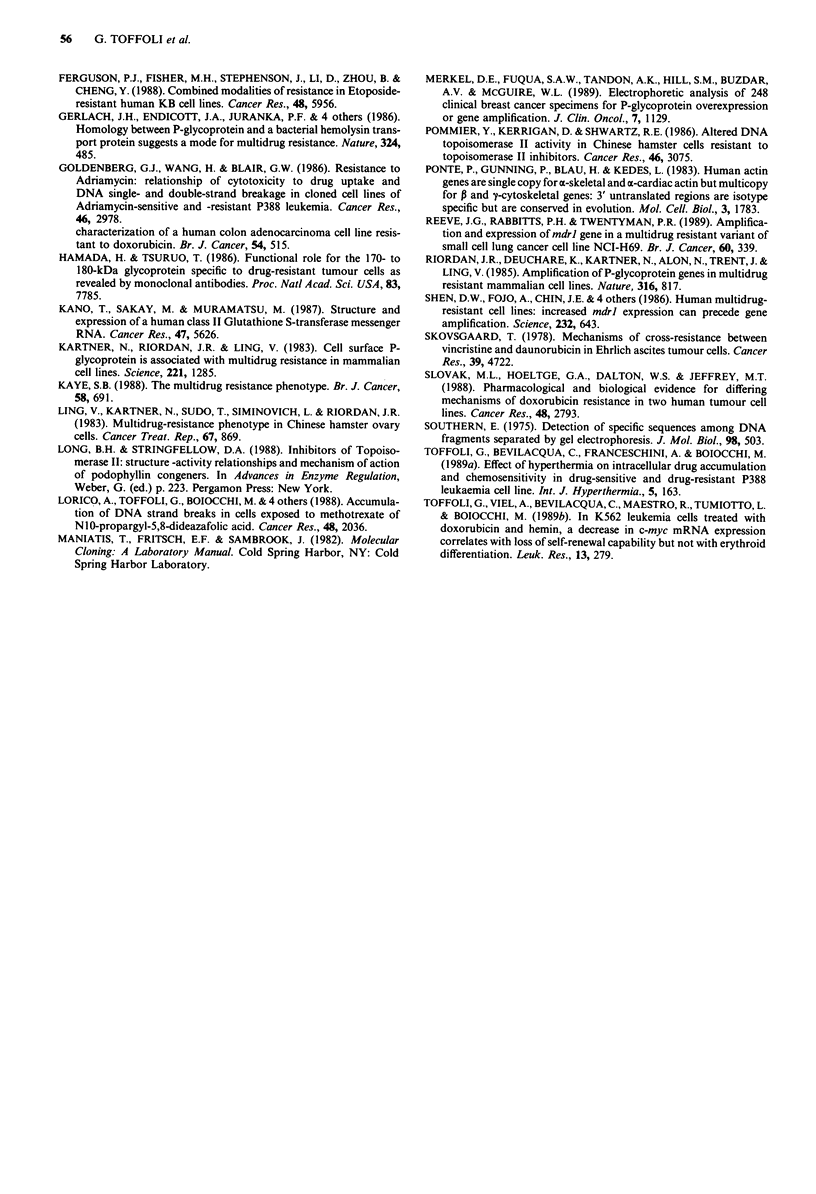# Pleiotropic-resistant phenotype is a multifactorial phenomenon in human colon carcinoma cell lines.

**DOI:** 10.1038/bjc.1991.11

**Published:** 1991-01

**Authors:** G. Toffoli, A. Viel, L. Tumiotto, G. Biscontin, C. Rossi, M. Boiocchi

**Affiliations:** Division of Experimental Oncology 1, Centro di Riferimento Oncologico, Aviano, Italy.

## Abstract

**Images:**


Br. J. Cancer (1991), 63, 51-56 © Macmillan Press Ltd., 1991

### Pleiotropic-resistant phenotype is a multifactorial phenomenon in human colon carcinoma cell lines

G. Toffoli[1], A. Viel[1], L. Tumiotto[1], G. Biscontin[1], C. Rossi[2] & M. Boiocchi[1]

[1]*Division of Experimental Oncology 1 and* [2]*Division of Surgery Oncology, Centro di Riferimento Oncologico, Via Pedemontana Occidentale, 33081 Aviano, Italy.*

**Summary** The biochemical basis of multidrug-resistant (MDR) phenotype has been investigated in drug-resistant sublines independently obtained in our laboratories by single step doxorubicin (DOX) selection of LoVo, DLD1, and SW948 human colon carcinoma (HCC) cell lines. All the chemoresistant sublines have been found to be cross-resistant to DOX, actinomycin-D (ACT-D) and vincristine (VCR) but not to cis-diamminedichloroplatinum (CDDP), and have exhibited an increased expression level of *mdr*1 mRNA and gp170 glycoprotein. Comparative analyses in drug-resistant and sensitive cells of resistance index, extracellular and intracellular equitoxic DOX concentrations, and *mdr*1 gene products expression have indicated that MDR phenotype is a multifactorial phenomenon due to different and possibly independent biochemical mechanisms which cooperate, in varying degrees from cell line to cell line, in conferring cellular chemoresistance.

*In vitro* derived drug-resistant cell lines frequently show a typical wide spectrum of chemoresistance to many structurally and functionally unrelated drugs (Ling *et al.*, 1983; Kaye, 1988). The multidrug-resistant (MDR) phenotype in human cells is thought to be primarily consequent upon the increased expression of the *mdr*1 gene (Riordan *et al.*, 1985; Shen *et al.*, 1986; Chin *et al.*, 1989) which encodes for a plasma transmembrane glycoprotein of 170 kD (gp 170) (Kartner *et al.*, 1983; Chen *et al.*, 1986). gp170 causes reduced intracellular drug accumulation through an energy-dependent active drug efflux (Dano, 1973; Skovsgaard, 1978). Recent studies, however, have reported that biochemical phenomena, unrelated to increase in gp170 expression and/or function, also contribute in conferring the MDR phenotype (Goldenberg *et al.*, 1986; Slovak *et al.*, 1988; Ferguson *et al.*, 1988; Cole *et al.*, 1989; Reeve *et al.*, 1989; Toffoli *et al.*, 1989*a*).

In order to define the molecular basis of the MDR phenotype in human colon carcinoma (HCC) cells, we have derived MDR sublines from LoVo, DLD1 and SW948 HCC cell lines. Five drug- resistant sublines independently obtained from each parent line have been characterised for: (1) resistance pattern; (2) extracellular (IC50ext) and intracelluar (IC50int) DOX concentration inhibiting cell growth of 50%; (3) DOX efflux kinetic; and (4) *mdr1* gene products expression.

### Materials and methods

### *Chemicals*

Doxorubicin [(DOX), Adriamicina, Farmitalia, Carlo Erba, Milan, Italy]; vincristine [(VCR), Oncovin, Lilly, Florence, Italy]; actinomycin D [(ACT-D), Cosmegen, Merck, Sharp, Rome, Italy]; cis-diamminedichloroplatinum [(CDDP), Platinex, Bristol, Latina, Italy] were sterilely dissolved in saline just before use. $^{14}$C-doxorubicin ($^{14}$C-DOX) (specific activity 55 mCi mmol$^{-1}$); D-1-$^{14}$C mannitol (specific activity 59 mCi mmol$^{-1}$) and $^{3}H_2O$ (specific activity 5 mCi ml$^{-1}$) were purchased from Amersham (Buckinghamshire, UK).

### *Cell lines*

LoVo, DLD1 and SW948 HCC cell lines were obtained from the American Type Culture Collection (Rockville, MD). Cell lines were propagated in F12 (LoVo) or RPMI 1640 (DLD1 and SW948) supplemented with 10% heat-inactivated fetal calf serum (FCS) (Seralab, Sussex, UK), 1 mM Napyruvate, 50 μg ml$^{-1}$ streptomycin and 50 units ml$^{-1}$ penicillin G. Cell cultures were incubated at 37°C in a humidified atmosphere of 5% $CO_2$ and 95% air.

### *Cytotoxicity tests*

Cytotoxic effects of pharmacological treatments were determined by clonogenic assay in liquid medium after about 10 days from drug exposure as described (Lorico *et al.*, 1988). No significant variations were noted for plating efficiencies in monolayer culture between untreated sensitive parent and drug-resistant cells. Experimental data obtained by clonogenic assay were also confirmed by the trypan blue dye exclusion test, 72 h from cell treatment.

Drug cytotoxicity was calculated as a percentage of cell survival in drug-treated cultures compared to that in untreated controls. Results are reported as drug concentration inhibiting cell growth of 50% (IC50). IC50 was extrapolated by linear regression of experimental data.

### *Selection of drug-resistant cell mutants*

Drug-resistant sublines were obtained by treating exponentially growing cell cultures in continuous with 100 ng ml$^{-1}$ DOX as described (Grandi *et al.*, 1986). The medium was replaced twice a week with fresh medium containing 100 ng ml$^{-1}$ DOX. After 5-10 weeks, single colonies of drug-resistant mutants were harvested with 0.25% trypsin/0.02% EDTA and singularly cultured for more than 12 months in the continuous presence of 100 ng ml$^{-1}$ DOX. Drug-resistant sublines were maintained in presence of 100 ng ml$^{-1}$ DOX until at least 24 h before the experiments.

### *Northern and Southern analyses*

Total cellular RNA and high molecular weight DNA were extracted from exponentially growing cells by the guanidine chloride method (Cox, 1968) and the proteinase/phenol-chloroform method (Maniatis *et al.*, 1982), respectively.

For Northern blots, RNAs were fractionated by electrophoresis in a denaturing 1% agarose/6% formaldehyde gel and transferred to Gene Screen Plus membrane (New England Nuclear, Florence, Italy) by electroblotting in 0.025 M phosphate buffer pH 6.5 (10 Volts overnight and 40 Volts for 1 h). For Southern blot analysis, 10 μg of DNA were digested with the EcoRI restriction endonucleases, electrophoresed in agarose gel and blotted onto a Gene Screen Plus membrane as described (Southern, 1975).

Filters were prehybridised, hybridised and washed as de-

Correspondence: M. Boiocchi, Experimental Oncology 1, Centro di Riferimento Oncologico, Via Pedemontana Occidentale, 33081 Aviano, Italy.
Received 3 May 1990; and in revised form 2 July 1990.

scribed (Toffoli *et al.*, 1989*b*) and exposed to X-ray films with an intensifying screen at −80°C. The autoradiographic signals were quantified by densitometry. Quantification of *mdr1* mRNA levels were performed using β-actin mRNA levels as internal standard. *mdr1* mRNA expression levels were reported in arbitrary units. A value of 10 units for the expression of 10 μg of total RNA from SW948 cells was assigned.

Probes used were: a 1.2 kb EcoRI fragment derived from plasmid pHuP170 (kindly provided by W.L. McGuire), representing a cDNA covering the 3′ portion of the human *mdr*1 gene (Merkel *et al.*, 1989), a human β-actin fragment (0.7 kb) derived from BamHI-EcoRI digested plasmid pHFβA-3′UT (Ponte *et al.*, 1983). Probes were $^{32}$P-labelled by multiprime labelling system (Amersham) at specific activity $>10^9$ counts min$^{-1}$ per 1 μg DNA.

### *Cytofluorimetric analysis*

gp170 expression was analysed by indirect immunofluorescence techniques: 5–10 × $10^5$ cells were fixed in 3.7% formaldehyde/PBS (phosphate buffered saline) for 10 min at 20°C and preincubated for 10 min at 20°C in 10% normal goat serum. MRK16 mouse monoclonal antibody (kindly provided by Dr Tsuruo) (Hamada & Tsuruo, 1986), diluted to 30 μg ml$^{-1}$ in PBS containing 1 mM EDTA and 0.1% saponin, was used as first antibody and FITC-conjugated goat antimouse IgG (Becton Dickinson, Mountain View, CA) diluted 1 : 20 in PBS/EDTA/saponin was used as second antibody. Labelled cells were analysed using a Becton Dickinson FACStar IV. Cells incubated with normal mouse IgGs as first antibody were used as negative control.

### *DOX-uptake analysis and determination of IC50int DOX*

Exponentially growing cells, 3 × $10^6$ in 10 ml medium, were seeded in 90 mm petri dishes (Falcon, Milan, Italy) and incubated overnight at 37°C. After removing culture medium, cells were incubated for 1.5 h at 37°C with fresh medium containing different concentrations of $^{14}$C-DOX. Radioactive medium was subsequently withdrawn and petri dishes were chilled on ice and quickly washed 3 times with ice-cold saline solution. Cells were harvested by trypsin treatment and counted using an emocytometer. $^{14}$C-DOX uptake was determined by liquid scintillation counting. Cytotoxic effect of drug treatments was determined on replicate petri dishes.

Cells size was determined using $^3H_2O$ and D-1-$^{14}$C mannitol as described (Long & Stringfellow, 1988). Intracellular drug concentration was calculated by dividing the intracellular drug content by the cell volume.

The intracellular DOX concentration inhibiting cell growth of 50% (IC50int DOX) was determined after exposing the cells for 1.5 h to the external drug concentration inhibiting cell growth of 50% (IC50ext DOX).

### *DOX-efflux analysis*

Exponentially growing cells, 3 × $10^6$ in 10 ml medium, were seeded in 90 mm petri dishes and incubated overnight at 37°C. After removal of culture medium, cells were incubated at 37°C for 1.5 h with fresh medium containing $^{14}$C-DOX. Following incubation the medium was discarded and the cells were quickly washed in a saline solution prewarmed at 37°C, then prewarmed DOX-free medium was added and the plates were incubated at 37°C. Retention of $^{14}$C-DOX in the cells was determined at various time points (1.5–60 min) using three petri dishes for each time point, as described for drug-uptake studies. Efflux data are expressed as percentages of the drug retained at each time point relative to drug retained at time point 0 min.

### *Statistic*

For the statistical evaluation of the data, the unpaired Student's test was used.

### Results

### *Chemosensitivity of parent-HCC cell lines and derived drug-resistant sublines*

Chemosensitivity of LoVo, DLD1 and SW948 parent-cell lines to DOX, VCR, ACT-D and CDDP has been determined both by clonogenic assay and trypan blue dye exclusion test. As shown in Table I, the 3 cell lines were almost equally sensitive to the different drugs tested.

From each parent line five independently derived DOX-resistant sublines were characterised for chemoresistance. All sublines exhibited the MDR phenotype and were more resistant than the parent line to DOX, VCR, ACT-D but not to CDDP (Table II). The increases in chemoresistance to DOX, VCR, ACT-D presented very little variations among the sublines derived from each parent line, but were consistently different among the 3 subline groups. DOX chemoresistance increased about 20 times in LoVo drug-resistant (LoVo-R), about 9 times in DLD1 drug-resistant (DLD1-R) and about 4 times in SW948 drug-resistant (SW948-R) sublines (Table II). SW948-R sublines showed a different profile of resistance to VCR and ACT-D with respect to DLD1-R and LoVo-R sublines. In fact, SW948-R sublines exhibited a relative greater increase in resistance to ACT-D than to VCR, whereas this pattern was inverted in LoVo-R and DLD1-R sublines (Table II).

### mdr*1 mRNA transcription level and gp170 expression*

Expression level of *mdr1* mRNA was determined by North-

**Table I** Sensitivity of the human colon carcinomas cell lines to various antitumour agents

| Cell line | DOX | ACT-D | VCR | CDDP |
|---|---|---|---|---|
| | *(IC50 ng ml$^{-1}$)*[a] | | | |
| SW948 | 23.36 (±2.71)[b] | 10.22 (±2.62) | 50.43 (±5.39) | 478.30 (±64.51) |
| | 22.87 (±1.18)[c] | 14.02 (±1.17) | 53.97 (±9.60) | 503.02 (±11.62) |
| DLD1 | 22.25 (±3.40) | 10.12 (±3.57) | 49.87 (±6.84) | 543.99 (±77.04) |
| | 23.86 (±3.91) | 12.97 (±3.82) | 56.31 (±8.97) | 642.98 (±59.24) |
| LoVo | 20.11 (±4.58) | 11.62 (±2.29) | 51.32 (±4.41) | 401.75 (±132.00) |
| | 25.29 (±2.04) | 13.25 (±4.73) | 57.75 (±6.88) | 469.75 (±63.02) |

[a]Extracellular dose inhibiting cell growth of 50% (IC50). Data are from at least three experiments, each done in triplicate. [b]The upper concentrations refer to clonogenic assay, [c]the lower ones to the trypan blue dye exclusion test. Cells were exposed to the drug for 24 h.

**Table II** Cross resistance pattern of MDR sublines with various antitumour agents

| Cell line | DOX | Relative resistance[a] ACT-D | VCR | CDDP |
|---|---|---|---|---|
| SW948 | 1 | 1 | 1 | 1 |
| SW948-R-1 | 4.2[b] | ND | 1.9[b] | ND |
| SW948-R-2 | 4.3[b] | 3.8[b] | 1.8[b] | 1.4 |
| SW948-R-3 | 3.2[b] | 3.9[b] | ND | ND |
| SW948-R-5 | 4.5[b] | 5.1[b] | 2.1[b] | 1.4 |
| SW948-R-6 | 4.5[b] | 4.9[b] | 2.0[b] | 1.5 |
| DLD1 | 1 | 1 | 1 | 1 |
| DLD1-R-2 | 8.6[b] | 2.8[b] | 4.1[b] | 1.2 |
| DLD1-R-4 | 8.8[b] | ND | ND | 1.1 |
| DLD1-R-5 | 9.4[b] | 3.2[b] | 4.7[b] | ND |
| DLD1-R-6 | 10.4[b] | 4.8[b] | 7.8[b] | 1.1 |
| DLD1-R-8 | 11.1[b] | 4.1[b] | 6.8[b] | 1.0 |
| LoVo | 1 | 1 | 1 | 1 |
| LoVo-R-1 | 22.7[b] | 8.6[b] | 14.3[b] | 0.82 |
| LoVo-R-2 | 20.0[b] | 8.2[b] | 11.1[b] | 0.64 |
| LoVo-R-3 | 22.5[b] | 7.3[b] | 15.3[b] | 0.84 |
| LoVo-R-4 | 21.1[b] | 5.9[b] | 12.6[b] | 1.02 |
| LoVo-R-5 | 19.3[b] | 8.0[b] | 10.3[b] | 0.98 |

[a]Relative resistance is the ratio of IC50 for resistant cells treated with a particular drug for 24 h to the IC50 for sensitive cells treated with the same agent for 24 h. Data were obtained from the mean of at least three experiments each done in triplicate, using the clonogenic assay. [b]Indicates when IC50 of resistant lines was statistically different, by Student's test, from IC50 of sensitive lines ($P<0.01$). ND, not determined.

ern analysis. SW948, DLD1 and LoVo cell lines expressed 10, 16 and 36 *mdr*1 mRNA units, respectively. *mdr1* mRNA expression level increased in all drug-resistant sublines by a roughly identical factor, becoming about 10 times greater than that expressed by the respective parent cell line (Figure 1). The increased *mdr*1 mRNA expression in all LoVo-R sublines may have been consequent upon gene amplification; each subline showed, in fact, 4–5 *mdr*1 gene copies per aploid genome (data not shown). DLD1-R and SW948-R sublines presented neither gene amplification nor rearrangement (data not shown).

**Figure 1** Northern hybridisation of RNA from SW948-R, DLD1-R, LoVo-R resistant clones and parent-cell lines. Each lane contains 10 μg of total cellular RNA. After *mdr1* hybridisation, filters were rehybridised with a human β-actin probe as control for RNA loading. *mdr1* mRNA units were calculated from the area under the densitometric peaks for the *mdr1* signals normalised by β-actin signals.

**Figure 2** Flow cytometric profiles of drug-resistant sublines. Shown is one representative chemoresistant subline (SW948-R-5; DLD1-R-6 and LoVo-R-1) obtained from SW948, DLD1 and LoVo parent-cell line, respectively. **A**, cells incubated with non-immune mouse IgGs as negative control and FITC-goat antimouse IgGs (Chemoresistant sublines gave similar results); **B** and C, cells incubated with MRK16 mouse monoclonal antibody and FITC-goat antimouse IgGs. The log fluorescence intensity was plotted on the x-axis against the relative cell number on the y-axis.

Cytofluorimetric analyses showed that the increased *mdr*1 mRNA expression levels observed in drug-resistant sublines were associated with a concomitant increase in gp170 expression (Figure 2).

### *Transmembrane drug equilibria and intracellular equitoxic drug concentrations*

Parent-cell lines and drug-resistant sublines were analysed for transmembrane DOX equilibria to ascertain whether the enhanced *mdr*1 gene expression in the MDR sublines might completely explain their increased drug resistance. Analyses were performed on 2, randomly chosen, MDR sublines for each parent-cell line: LoVo-R-1 and LoVo-R-2, DLD1-R-6 and DLD1-R-8, SW948-R-5 and SW948-R-6. Intracellular DOX accumulation was determined after 1.5 h of continuous cell exposure to the drug, at which time the steady-state in transmembrane drug equilibria was completely established (data not shown).

As shown in Figure 3, after exposure to the same extracellular DOX concentrations, the corresponding intracellular drug concentrations were very similar within the 3 parent-cell lines and SW948-R sublines. On the contrary, DOX accumulations decreased by about 50–60% in DLD1-R and 60–70% in LoVo-R sublines, as compared to the parent cells.

To determine whether the MDR phenotype in drug-resistant sublines is consequent upon increased intracellular resistance to the drug, the intracellular DOX concentration causing a 50% inhibition of cell growth (IC50int DOX) was established. The three parent-cell lines, that had a similar chemosensitivity to extracellular DOX concentrations (Table I), displayed also a similar intracellular sensitivity to the drug (Table III). In contrast, although the extracellular drug concentrations inhibiting cell growth of 50% (IC50ext DOX) were different among the three subsets MDR sublines, their respective IC50int DOX were similar. All MDR sublines exhibited, in fact, an IC50int DOX about 3.5 times greater than the IC50int DOX of parent-cell lines (Table III). Therefore the intracellular drug resistance of the three groups of MDR sublines increased uniformly, in spite of different *mdr*1 mRNA expression levels exhibited. This property was also confirmed in a wide range of intracellular drug concentrations (Figure 4).

**Table III** Extracellular and intracellular DOX concentrations inhibiting cell growth of 50% (IC50) in sensitive and resistant cells

| *Cell line* | *IC50ext DOX*[a] *(ng ml$^{-1}$)* | *IC50int DOX*[b] *(pmol DOX $10^{-6}$ cells)* | *Res/Sens* |
|---|---|---|---|
| SW948 | 350 | 146.73±21.32 | 1 |
| SW948-R-5 | 1400 | 547.57±54.51 | 3.73 |
| SW948-R-6 | 1400 | 581.20±127.36 | 3.96 |
| DLD1 | 350 | 160.23±15.87 | 1 |
| DLD1-R-6 | 3400 | 553.66±94.93 | 3.46 |
| DLD1-R-8 | 3600 | 579.95±87.10 | 3.62 |
| LoVo | 350 | 171.30±18.04 | 1 |
| LoVo-R-1 | 7500 | 625.28±105.64 | 3.65 |
| LoVo-R-2 | 6600 | 600.34±80.36 | 3.50 |

[a]Extracellular DOX concentration inhibiting cell growth of 50% after 1.5 h exposure to DOX. Each value represents the mean ± s.d. of at least five independent experiments. [b]Corresponding intracellular DOX concentration (IC50int DOX).

$$\text{RES/SENS: } \frac{\text{IC50int resistant subline}}{\text{IC50int sensitive parent-cell line}}$$

### *DOX-efflux kinetic*

Efflux kinetic analyses performed on drug-sensitive parent-cell lines and drug-resistant sublines indicated that decreases of intracellular DOX accumulation in DLD1-R and LoVo-R sublines were consequent upon an increased drug efflux (Figure 5). Since the metabolic inhibitor Na azide eliminated the differences in transmembrane drug equilibria observed (data not shown), it is assumed that drug efflux was due to an energy-dependent biochemical mechanism. No significant differences in DOX efflux were observed among SW948, DLD1 and LoVo drug-sensitive and SW948-R drug-resistant cell lines.

### Discussion

The present study has indicated that MDR phenotype, at least in the HCC cell lines studied, is a multifactorial phenomenon determined by different and possibly independent biochemical mechanisms which cooperate, in amounts varying from cell line to cell line, in conferring cellular chemoresistance. The analyses on transmembrane DOX equilibria

**Figure 3** Intracellular DOX concentrations in SW948, DLD1 and LoVo sensitive and resistant cells. Intracellular DOX accumulations were determined after treating cells for 1.5 h with the extracellular DOX concentrations indicated on the right of the figure. Intracellular drug concentrations (expressed as pmol DOX $10^{-6}$ cells) were obtained by normalising DOX accumulations for the cell size of DLD1 sensitive cell line at which a value of 1 was assigned. Ratios between the size of DLD1 sensitive cell line and SW948, SW948-R-5 and SW948-R-6 were 1.21, 0.89 and 0.91, respectively; the ratios between DLD1 and DLD1-R-6 and DLD1-R-8 were 1.07 and 1.10, respectively; the ratios between DLD1 and LoVo, LoVo-R-1 and LoVo-R-2 were 0.87, 0.83 and 0.80, respectively. *indicates when intracellular DOX concentrations in resistant sublines were significatively different from the parent cell line ($P<0.01$) after treatment with the same extracellular DOX concentration. Bars, s.d.

**Figure 4** Effect of intracellular DOX concentration on cell survival of SW948-R-5 (◇), DLD1-R-6 (△) and LoVo-R-1 (□) sublines. Points, mean from 4 to 7 determinations.

**Figure 5** Efflux of $^{14}$C-DOX from sensitive and resistant cells. ◇ SW948-R-5, △ DLD1-R-6, □ LoVo-R-1 cell lines; dark symbols: parent cell lines; open symbols: resistant sublines. Data are referred as percentage of DOX retained in cells with respect to time 0 (100%). Points, mean of five experiments. A statistically significant difference between resistant and parent cell lines is indicated in the figure with asterisks (*$P<0.05$; **$P<0.01$).

and equitoxic intracellular DOX accumulations have shown that the biochemical phenoma, which determine multidrug-resistance, exert their effect on different cellular compartments. The first mechanism acts at the plasma-membrane level and affects drug transmembrane equilibria determining reduced intracellular drug accumulation. Said mechanism may be ascribed to the biochemical activity of the *mdr*1 gene product, the gp170 (Chen *et al.*, 1986), as indicated by the DOX efflux and *mdr*1 gene expression studies in LoVo-R and DLD1-R sublines. With respect to the parent-cell lines, both LoVo-R and DLD1-R variants showed, in fact, increased *mdr*1 gene transcriptional and translational activities determining an increased DOX efflux and reduced drug uptake in the cells. This suggests that in LoVo-R and DLD1-R sublines, higher levels of *mdr*1 gene products confer a selective advantage during drug exposure. On the contrary, we cannot at present explain the biological meaning of enhanced *mdr*1 mRNA and gp170 expression observed in SW948-R sublines. We have been unable to determine any reduction in intracellular DOX accumulation even when SW948-R sublines were treated with the extracellular DOX concentration used for their selection from the parent line (100 ng ml$^{-1}$). This suggests that relatively low levels of *mdr*1 expression, such as those displayed by the three parent-cell lines and SW948-R sublines, do not affect drug-transmembrane equilibria. Alternatively, variations in drug-transmembrane equilibria in SW948-R might be smaller than in LoVo-R or DLD1-R variants and not measurable with our experimental procedures (Deffie *et al.*, 1988), or the *mdr*1 basic function (still undefined) in parent cells may be augmented in SW948-R variants without affecting drug-transmembrane transport, as suggested by Gerlach *et al.* (1986).

The second mechanism, which confers drug-resistance to the HCC MDR sublines exerts its effect at intracellular level, as evidenced by the greater intracellular DOX concentration needed to obtain a 50% cell growth inhibition (IC50int DOX) in all drug-resistant sublines investigated. This finding implies that biochemical mechanisms, independent of transmembrane drug equilibria, increase their effect in MDR sublines. Our data strongly support previously reported results on MDR small lung cancer cell lines obtained by Cole *et al.* (1989) and Reeve *et al.* (1989) which also indicated that at least two different biochemical phenomena lead to MDR. The first one involves gp170 whereas the second is not at present identifiable at molecular level. Possibly, this second drug-resistance mechanism is the only active one in SW948-R sublines. Two gene systems are presently thought to be related to intracellular drug resistance: topoisomerases (Pommier *et al.*, 1986) and glutathione-S-transferase (Kano *et al.*, 1987). These cellular enzymes are involved in DNA conformational modification and cellular detoxification, respectively. The peculiar pattern of cross resistance presented by SW948-R sublines may be useful in elucidating which of these gene systems, if any, is implicated in the intracellular drug-resistance mechanism.

Finally, in all drug-resistant sublines analysed, each drug-resistance mechanism increased its effect, as compared to the parent cell line, by a specific and constant factor. In fact, *mdr*1 gene expression level increased about 10 times and the intracellular drug resistance about 3.5 times. At present, we do not know the molecular basis of such a phenomenon, however the constancy of the factorial occurrence looks very promising and deserving of further studies.

We thank Dr William L. McGuire for providing the *mdr1* gene cDNA probe, Dr Takashi Tsuruo for MRK16 monoclonal antibody. We also thank Mrs Marisa Caruso for helping with the English version, Paolo Callegari for technical assistance and Miss Paola Pistello for her help with the manuscript.

This study was supported by a grant from the Italian Association for Cancer Research, Milan, Italy and CNR Progetto Finalizzato 'Oncologia', contract N. 88.00537.44.

### References

CHEN, C., CHIN, J.E., UEDA, K. & 4 others (1986). Internal duplication and homology with bacterial transport proteins in the *mdr1* (P-glycoprotein) gene from multidrug-resistant human cells. *Cell*, **47**, 381.

CHIN, J.E., SOFFIR, R., NOONAN, K.E., CHOI, K. & RONINSON, I.B. (1989). Structure and expression of the human MDR (P-glycoprotein) gene family. *Mol. Cell. Biol.*, **9**, 3808.

COLE, S.P.C., DOWNES, H.F. & SLOVAK, M.L. (1989). Effect of calcium antagonists on the chemosensitivity of two multidrug-resistant human tumour cell lines which do not overexpress P-glycoprotein. *Br. J. Cancer*, **59**, 42.

COX, R.A. (1968). The use of guanidine chloride in the isolation of nucleic acid. *Meth. Enzymol.*, **12**, 120.

DANO, K. (1973). Active outward transport of daunomycin in resistant Ehrlich ascites tumour cells. *Biochem. Biophys. Acta*, **323**, 446.

DEFFIE, A.M., ALAM, T., SENEVIRATNE, C. & 5 others (1988). Multifactorial resistance to Adriamycin: relationship of DNA repair, glutathione transferase activity, drug efflux, and P-glycoprotein in cloned cell lines of Adriamycin-sensitive and -resistant P388 leukemia. *Cancer Res.*, **48**, 3595.

FERGUSON, P.J., FISHER, M.H., STEPHENSON, J., LI, D., ZHOU, B. & CHENG, Y. (1988). Combined modalities of resistance in Etoposide-resistant human KB cell lines. *Cancer Res.*, **48**, 5956.

GERLACH, J.H., ENDICOTT, J.A., JURANKA, P.F. & 4 others (1986). Homology between P-glycoprotein and a bacterial hemolysin transport protein suggests a mode for multidrug resistance. *Nature*, **324**, 485.

GOLDENBERG, G.J., WANG, H. & BLAIR, G.W. (1986). Resistance to Adriamycin: relationship of cytotoxicity to drug uptake and DNA single- and double-strand breakage in cloned cell lines of Adriamycin-sensitive and -resistant P388 leukemia. *Cancer Res.*, **46**, 2978.

characterization of a human colon adenocarcinoma cell line resistant to doxorubicin. *Br. J. Cancer*, **54**, 515.

HAMADA, H. & TSURUO, T. (1986). Functional role for the 170- to 180-kDa glycoprotein specific to drug-resistant tumour cells as revealed by monoclonal antibodies. *Proc. Natl Acad. Sci. USA*, **83**, 7785.

KANO, T., SAKAY, M. & MURAMATSU, M. (1987). Structure and expression of a human class II Glutathione S-transferase messenger RNA. *Cancer Res.*, **47**, 5626.

KARTNER, N., RIORDAN, J.R. & LING, V. (1983). Cell surface P-glycoprotein is associated with multidrug resistance in mammalian cell lines. *Science*, **221**, 1285.

KAYE, S.B. (1988). The multidrug resistance phenotype. *Br. J. Cancer*, **58**, 691.

LING, V., KARTNER, N., SUDO, T., SIMINOVICH, L. & RIORDAN, J.R. (1983). Multidrug-resistance phenotype in Chinese hamster ovary cells. *Cancer Treat. Rep.*, **67**, 869.

LONG, B.H. & STRINGFELLOW, D.A. (1988). Inhibitors of Topoisomerase II: structure -activity relationships and mechanism of action of podophyllin congeners. In *Advances in Enzyme Regulation*, Weber, G. (ed.) p. 223. Pergamon Press: New York.

LORICO, A., TOFFOLI, G., BOIOCCHI, M. & 4 others (1988). Accumulation of DNA strand breaks in cells exposed to methotrexate of N10-propargyl-5,8-dideazafolic acid. *Cancer Res.*, **48**, 2036.

MANIATIS, T., FRITSCH, E.F. & SAMBROOK, J. (1982). *Molecular Cloning: A Laboratory Manual.* Cold Spring Harbor, NY: Cold Spring Harbor Laboratory.

MERKEL, D.E., FUQUA, S.A.W., TANDON, A.K., HILL, S.M., BUZDAR, A.V. & MCGUIRE, W.L. (1989). Electrophoretic analysis of 248 clinical breast cancer specimens for P-glycoprotein overexpression or gene amplification. *J. Clin. Oncol.*, **7**, 1129.

POMMIER, Y., KERRIGAN, D. & SHWARTZ, R.E. (1986). Altered DNA topoisomerase II activity in Chinese hamster cells resistant to topoisomerase II inhibitors. *Cancer Res.*, **46**, 3075.

PONTE, P., GUNNING, P., BLAU, H. & KEDES, L. (1983). Human actin genes are single copy for α-skeletal and α-cardiac actin but multicopy for β and γ-cytoskeletal genes: 3′ untranslated regions are isotype specific but are conserved in evolution. *Mol. Cell. Biol.*, **3**, 1783.

REEVE, J.G., RABBITTS, P.H. & TWENTYMAN, P.R. (1989). Amplification and expression of *mdr1* gene in a multidrug resistant variant of small cell lung cancer cell line NCI-H69. *Br. J. Cancer*, **60**, 339.

RIORDAN, J.R., DEUCHARE, K., KARTNER, N., ALON, N., TRENT, J. & LING, V. (1985). Amplification of P-glycoprotein genes in multidrug resistant mammalian cell lines. *Nature*, **316**, 817.

SHEN, D.W., FOJO, A., CHIN, J.E. & 4 others (1986). Human multidrug-resistant cell lines: increased *mdr1* expression can precede gene amplification. *Science*, **232**, 643.

SKOVSGAARD, T. (1978). Mechanisms of cross-resistance between vincristine and daunorubicin in Ehrlich ascites tumour cells. *Cancer Res.*, **39**, 4722.

SLOVAK, M.L., HOELTGE, G.A., DALTON, W.S. & JEFFREY, M.T. (1988). Pharmacological and biological evidence for differing mechanisms of doxorubicin resistance in two human tumour cell lines. *Cancer Res.*, **48**, 2793.

SOUTHERN, E. (1975). Detection of specific sequences among DNA fragments separated by gel electrophoresis. *J. Mol. Biol.*, **98**, 503.

TOFFOLI, G., BEVILACQUA, C., FRANCESCHINI, A. & BOIOCCHI, M. (1989*a*). Effect of hyperthermia on intracellular drug accumulation and chemosensitivity in drug-sensitive and drug-resistant P388 leukaemia cell line. *Int. J. Hyperthermia.*, **5**, 163.

TOFFOLI, G., VIEL, A., BEVILACQUA, C., MAESTRO, R., TUMIOTTO, L. & BOIOCCHI, M. (1989*b*). In K562 leukemia cells treated with doxorubicin and hemin, a decrease in c-*myc* mRNA expression correlates with loss of self-renewal capability but not with erythroid differentiation. *Leuk. Res.*, **13**, 279.